# Supplementary material for: Effectiveness and Safety of Ayurvedic Medicines in Type 2 Diabetes Mellitus Management: A Systematic Review and Meta-Analysis
Source: Front Pharmacol. 2022 Jun 8;13:821810. doi: 10.3389/fphar.2022.821810 (PMC9213670; doi:10.3389/fphar.2022.821810)
Supplement: Supplementary file 1 [file DataSheet1.zip › Supplementary Material/Forest and Funnel Plots/Cinnamomum aromaticum/LDL-C.pdf]

Cinnamomum aromaticum - LDL-C

Study	Intervention N	Intervention Mean	Intervention SD	Comparator N	Comparator Mean	Comparator SD	Mean Diff. with 95% CI	Weight (%)
Mang 2006	33	136.10	29.00	32	139.19	24.75	-3.09 [-16.22, 10.04]	14.10
Blevins 2007	29	-2.00	3.10	28	-3.00	5.60	1.00 [-1.34, 3.34]	27.08
Akilen 2010	30	97.44	38.67	28	90.48	30.16	6.96 [-10.98, 24.90]	9.87
Wainstein 2011	29	84.54	39.30	30	89.80	27.40	-5.26 [-22.50, 11.98]	10.38
Lu 2012	46	109.62	25.27	20	107.49	26.29	2.13 [-11.30, 15.56]	13.78
Sharma 2012	100	-12.41	55.92	50	22.30	61.73	-34.71 [-54.37, -15.05]	8.73
Tangvarasittichai 2015, Sengsuk 2016	49	85.84	24.92	50	83.90	32.36	1.94 [-9.45, 13.33]	16.06
**Overall**							-2.45 [-9.46, 4.56]	

Heterogeneity: $\tau^2 = 45.76$, $I^2 = 60.26\%$, $H^2 = 2.52$

Test of $\theta_i = \theta_j$: Q(6) = 13.83, p = 0.03

Test of $\theta = 0$: z = -0.69, p = 0.49

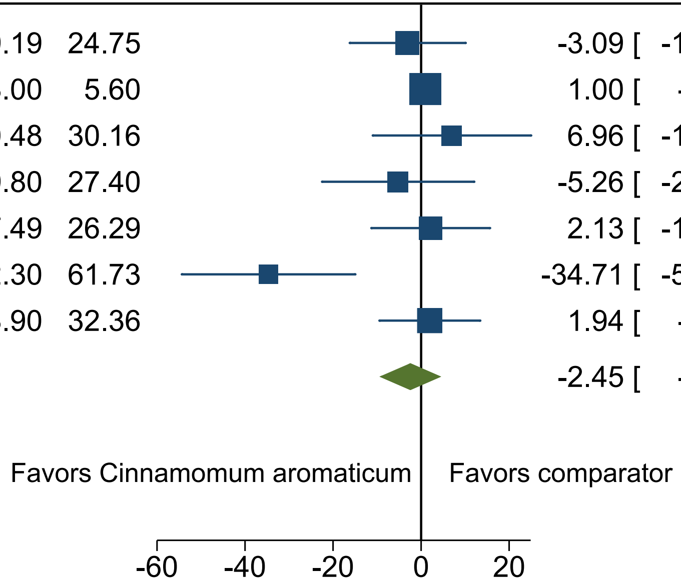

Random-effects REML model
